# Finger-Vein Recognition Using Heterogeneous Databases by Domain Adaption Based on a Cycle-Consistent Adversarial Network

**DOI:** 10.3390/s21020524

**Published:** 2021-01-13

**Authors:** Kyoung Jun Noh, Jiho Choi, Jin Seong Hong, Kang Ryoung Park

**Affiliations:** Division of Electronics and Electrical Engineering, Dongguk University, 30 Pildong-ro, 1-gil, Jung-gu, Seoul 04620, Korea; nohkyungjun@dongguk.edu (K.J.N.); choijh1027@dongguk.edu (J.C.); turtle1990@dgu.ac.kr (J.S.H.)

**Keywords:** finger-vein recognition, camera position, finger position, lighting, unobserved database, heterogeneous database, domain adaptation, cycle-consistent adversarial networks, SDUMLA-HMT-DB, HKPolyU-DB

## Abstract

The conventional finger-vein recognition system is trained using one type of database and entails the serious problem of performance degradation when tested with different types of databases. This degradation is caused by changes in image characteristics due to variable factors such as position of camera, finger, and lighting. Therefore, each database has varying characteristics despite the same finger-vein modality. However, previous researches on improving the recognition accuracy of unobserved or heterogeneous databases is lacking. To overcome this problem, we propose a method to improve the finger-vein recognition accuracy using domain adaptation between heterogeneous databases using cycle-consistent adversarial networks (CycleGAN), which enhances the recognition accuracy of unobserved data. The experiments were performed with two open databases—Shandong University homologous multi-modal traits finger-vein database (SDUMLA-HMT-DB) and Hong Kong Polytech University finger-image database (HKPolyU-DB). They showed that the equal error rate (EER) of finger-vein recognition was 0.85% in case of training with SDUMLA-HMT-DB and testing with HKPolyU-DB, which had an improvement of 33.1% compared to the second best method. The EER was 3.4% in case of training with HKPolyU-DB and testing with SDUMLA-HMT-DB, which also had an improvement of 4.8% compared to the second best method.

## 1. Introduction

Finger-vein images are difficult to forge and easy to obtain, but the image qualities are easily affected by the shades inevitably generated by other biological tissues (e.g., bone and fingernail) [1,2]. A finger-vein recognition system employs a small amount of feature for recognition because of this fundamental characteristic of data [3]. Therefore, models trained using such a dataset are ineffective for unobserved data. 

To consider this issue, non-training-based finger-vein recognition methods have been studied extensively to overcome this drawback. However, they exhibit significantly poorer performance than training-based methods because a large amount of information is removed by noise, thus making the classifier incapable of making an accurate decision [1,2,3]. Moreover, variations in the environment when acquiring images such as the camera position, lighting position, and lighting intensity create a large discrepancy between each dataset domain. This also deteriorates the performance of non-training-based methods. 

The existing non-training-based finger-vein recognition method extracts specific features using local binary patterns for recognition [1]; however, these features are significantly affected by misalignment or image quality, making them unsuitable for finger-vein recognition. Subsequently, local directional patterns (LDPs) [2] and optimal filter-based finger-vein recognition [3] have been proposed, which can solve the misalignment problem but cannot solve the fundamental problem of image quality or removed information. 

Hence, training-based finger-vein recognition methods have been extensively researched [4,5]. In [4], authors increased the number of training images by five times based on the data augmentation of image translation and cropping. In [5], they also increased the number of training images by 121 times based on the data augmentation of image translation and cropping. Although the similarity among augmented images increased by simple image translation and cropping, the training of proposed models was successfully performed with the augmented images, and the consequent accuracies of recognition were enhanced in their methods [4,5]. These methods exhibit good performance for finger-vein images with low quality by extracting features using a filter optimized for the distribution of input data rather than extracting features of a fixed form. 

Although the training-based methods exhibit better recognition performance than non-training-based methods, their recognition rate in the cross-domain environment is significantly lower. The training-based methods are trained for optimizing the distribution of the training data used as input; thus, they exhibit poorer generality in the cross-domain than non-training-based methods, which extract features of a fixed form regardless of the training data. Moreover, the distances between domains are inevitably increased as training is repeated with a small amount of information of finger-vein data. In general, a specific dataset used for training refers to one domain, and the model trained using this dataset is optimized for this specific domain. However, if the dataset of a different domain is used for testing, the performance is significantly deteriorated because the data encountered by the model are different from those used to train the model (the problem of heterogeneity). 

For mitigating the trade-off between recognition performance and generality, this study proposes a method for improving the finger-vein recognition rate of cross-domain databases through finger-vein domain adaptation using cycle-consistent adversarial networks (CycleGAN).

This paper is organized as follows. Section 2 presents previous studies related to the finger-vein recognition method, domain transfer, and domain adaptation, and Section 3 presents the contributions of this study. Section 4 provides the details of the proposed method, and Section 5 and Section 6 present the experimental results of this study and discussions, respectively. Lastly, Section 7 concludes this study.

## 2. Related Work

Research on finger-vein recognition in which domain adaptation is considered is lacking. In this section, the scope of previous studies is expanded to include hand-based biometrics; whether domain adaptation was performed, was analyzed by dividing the studies into non-training-based and training-based methods. 

### 2.1. Non-Training-Based Methods

Lu et al. performed domain adaptation to some extent by reducing the difference in brightness present in each finger-vein dataset using a peak-value-based method (PVM) [6]. The difference in brightness occurs when different sensors are used during acquisition of the dataset; this study focused on the difference between domains from this perspective. Jia et al. attempted to solve the cross-sensor problem using various dimension reduction algorithms and orientation coding methods [7]. 

Wang et al. performed a simple normalization to reduce the heterogeneity between domains for a dorsal hand-vein database obtained from various sensors and then performed segmentation to remove unimportant information which could increase heterogeneity [8]. In this study, matching was based on the scale-invariant feature transform (SIFT). The generality was high because matching was performed using a non-training-based algorithm; however, the performance was not suitable for biometric systems which require a high level of security. Wang et al. then performed soft domain adaptation using the same normalization algorithm followed by matching using an improved SIFT algorithm. This model was a more general and robust dorsal hand-vein recognition system [9].

Alshehri et al. used various handcrafted features to solve the problem of heterogeneity generated by different sensors when acquiring a fingerprint dataset, and in particular, ridge pattern, orientation, and minutiae points present in fingerprint images were used [10]. Binary gradient pattern (BGP) and Gabor-histogram of oriented gradients (Gabor-HoG) were used as descriptors, and the Sobel operator was used to compute the gradient. A robust fingerprint recognition system was proposed by performing score level fusion of the scores obtained from each descriptor. Ghiani et al. confirmed the problem with the accuracy of a fingerprint spoof attack detection system being abruptly reduced in the cross-sensor environment [11]. A least squares-based domain transformation function was adopted to reduce the extent of changes in the distribution caused by cross-sensors.

### 2.2. Training-Based Methods

Kute et al. used the Bregman divergence regularization method to reduce the distribution gap between domains; the researchers used Fisher linear discriminant analysis (FLDA) subspace learning algorithm to find a subspace through a projection matrix between fully heterogeneous data and then used the subspace to perform recognition using a support vector machine and K-nearest neighbor classifier [12]. Gajawada et al. performed domain adaptation between spoof attack databases to perform augmentation to improve the generality of a fingerprint spoof attack detector [13]. Here, a synthetic spoof attack patch was created using a universal material translator wrapper. 

Anand et al. customized the DeepDomainPore network, which is a pore detection network trained with high-resolution images to enable the pore information observed only in high-resolution fingerprint images to be used in low-resolution images [14]. Domain adaptation was performed for inserting pore information in the low-resolution image. Using this method, pores, which are a level 3 feature, can be exploited even when low-resolution images are input in a fingerprint recognition system. Shao et al. proposed PalmGAN, which generates synthetic data using a palmprint dataset with labels [15]. Fake labeled data were generated using the palmprint dataset without labels as the target and the palmprint dataset with labels as the source. The fake labeled data were then used as new data with a newly inserted label while maintaining the identity information of the target domain, i.e., domain adapted data. The data were input to a deep hash network to perform palmprint recognition. 

Moreover, the researchers attempted to solve the cross-domain problem by performing domain adaptation using an auto-encoder structured model [16]. Malhotra et al. highlighted the need to reinforce the touch-based biometric recognition system as the coronavirus disease (COVID-19) is increasingly becoming a serious issue across the globe [17]. Accordingly, the system was reinforced so that the fingerprint authentication system implements matching using a finger-selfie image. The finger-selfie image is segmented primarily using a handcrafted method to reduce the difference between the enrolled finger-scan image and finger-selfie domain. The segmented finger-selfie image and enrolled image undergo feature extraction through a deep ScatNet to allow matching with the trained random decision forest (RDF) model. 

Jalilian et al. performed finger-vein segmentation using a fully convolutional network (FCN) [18]. The recognition performance was assessed in the cross-domain environment using the segmented image. However, the performance was not satisfactory in the cross-domain environment even when recognition was performed using only compact information. Dabouei et al. verified the performance in the cross-sensor environment using a conditional generative adversarial network (CGAN) for fingerprint ridge map reconstruction [19]. 

Nogueira et al. performed fingerprint spoof attack detection using visual geometry group (VGG)-16 and a convolutional neural network (CNN) and verified that a deep learning-based method is not effective in the cross-data, cross-sensor environment, even though this study was not related to recognition [20]. Chugh et al. confirmed that fingerprint spoof detection based on the minutiae-based local patch approach and MobileNet did not exhibit good performance in the cross-sensor environment [21]. Thus, training the distribution of the training data in the cross-domain, cross-sensor environment without using specific domain adaptation methods is ineffective for unobserved databases. 

Although it is not the hand-based biometrics, Chui et al. proposed a CGAN and improved fuzzy c-means clustering (IFCM) algorithm called CGAN-IFCM for the multi-class voice disorder detection of three common types of voice disorders for smart healthcare applications [22].

To overcome the drawbacks of previous studies, we propose a method to improve the finger-vein recognition rate in cross-domain databases through finger-vein domain adaptation based on a CycleGAN. The reason for using CycleGAN in our method is that there is no paired data of input and target images in our experiments. That is, two finger-vein images from two different open databases (Shandong University homologous multi-modal traits finger-vein database (SDUMLA-HMT-DB) and Hong Kong Polytech University finger-image database (HKPolyU-DB)) are respectively used in our experiments. Because they are not from same class, there is no target image about input image in our case, and one of them can be used as input and the other can only be used as the reference image for the unpaired cases. Due to this reason, we use CycleGAN, which can use this kind of unpaired images. It is different from other types of GAN such as conditional GAN, which requires the paired data of input and target images [23]. 

CycleGAN can perform a task where the information of the source domain data is retained to some extent while reflecting the target domain information, instead of carrying out a task for simply making the source and target identical [24]. It is confirmed that our CycleGAN-based method showed better performances than other types of GAN.

## 3. Contributions

Our research is novel in the following five ways compared to previous works:

- This is the first study to examine GAN-based domain adaptation to solve the problem of performance deterioration of the finger-vein recognition system in a heterogeneous cross dataset.
- Domain adaptation was performed through a CycleGAN so that the existing training-based finger-vein recognition method can handle unobserved data. Each finger-vein dataset has different numbers of classes. Therefore, we used CycleGAN, which can deal with unpaired datasets.
- The proposed finger-vein recognition system does not have to be trained again when unobserved data are input into the system.
- The experiments with two open databases of SDUMLA-HMT-DB and HKPolyU-DB showed that the equal error rate (EER) of finger-vein recognition was 0.85% in case of training with SDUMLA-HMT-DB and testing with HKPolyU-DB, which is the improvement of 33.1% compared to the second best method. The EER was 3.4% in case of training with HKPolyU-DB and testing with SDUMLA-HMT-DB, which is also the improvement of 14.1% compared to the second best method.
- CycleGAN-based domain adaptation models and finger-vein recognition models trained with our domain adapted dataset proposed in this study are disclosed for a fair assessment of performance [25] by other researchers. On the website (http://dm.dgu.edu/link.html) explained in [25], we include the instructions of how other researchers can obtain our CycleGAN-based domain adaptation models and finger-vein recognition models.

## 4. Proposed Method

In this section, we would explain the overview of the proposed method in Section 4.1, our preprocessing method in Section 4.2, and proposed data adaption method based on CycleGAN in Section 4.3. In addition, we would explain the method of generating composite image for the input to CNN in Section 4.4, and finger-vein recognition method by DenseNet and shift matching in Section 4.5.

### 4.1. Overview of the Proposed Method

**Figure 1:**
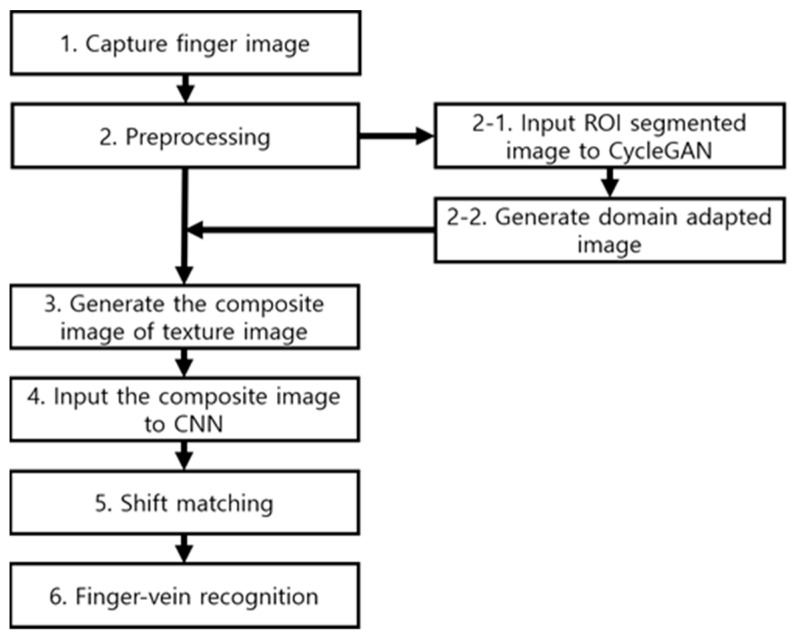
Overall procedure for the proposed finger-vein recognition method.

Figure 1 shows the overall procedure of the proposed finger-vein recognition method. The method involves preprocessing to remove unnecessary information generated by near-infrared light (NIR) used while acquiring images of finger veins, other biological tissues (e.g., bone or fingernail), or parts where information has been removed by shades [26] (Step 2 of Figure 1).

First, binary thresholding is performed to distinguish the finger region from the background region. The image that has undergone binary thresholding is used as a mask of the original finger-image and then undergoes linear stretching to fit the input size of a CNN subsequently. The finger region is not stretched uniformly if burrs are present in the mask during linear stretching. Thus, boundary smoothing enables the finger region to be stretched uniformly, thus minimizing information loss. 

In addition, misalignment may occur when the user’s finger trembles or is not fixed properly when acquiring finger-vein images. Misalignment is a major factor that reduces the finger-vein recognition performance. Hence, in-plane rotation compensation is performed to eliminate the misalignment problem. During in-plane rotation compensation, second-order moments of the entire image are found with respect to the finger-shape, and then, rotation is performed accordingly. In general, both edges of the finger image are thick and thus are affected more by biological tissues than other regions, or shades are generated by fingernails. Therefore, it is difficult to obtain the essential information of the finger vein. To overcome this problem, the parts are removed in the preprocessing step. Only the regions with the best finger-vein representation are segmented using the final mask obtained to be used as an input for finger-vein recognition.

The existing finger-vein recognition system has improved the performance of finger-vein recognition while being biased to the training dataset. The proposed method, in contrast, adds a domain adaptation stage to the acquired finger-vein images using a CycleGAN to better handle unobserved data, thus improving the generality of the finger-vein recognition system. After inputting the actual finger-vein images obtained in the preprocessing stage to the CycleGAN, the mapping function needed for domain adaptation is found during training. The mapping function converts the source domain into the target domain. Owing to the unpaired trait of the CycleGAN, a completely one-to-one mapping function is not observed; instead, training is continued to identify style information of the target domain. Therefore, the main structure of the data of the source domain is fairly maintained to create a new image to which the distribution characteristics of the target domain are transferred (Steps 2-1 and 2-2 of Figure 1). This process mitigates the heterogeneity between datasets.

Subsequently, a composite image is generated using the new image obtained with a CycleGAN (Step 3 of Figure 1), and it is then input to a densely connected network (DenseNet)-161 (Step 4 of Figure 1). Then, finger-vein recognition is finally performed via shift matching (Steps 5 and 6 of Figure 1).

### 4.2. Preprocessing 

**Figure 2:**
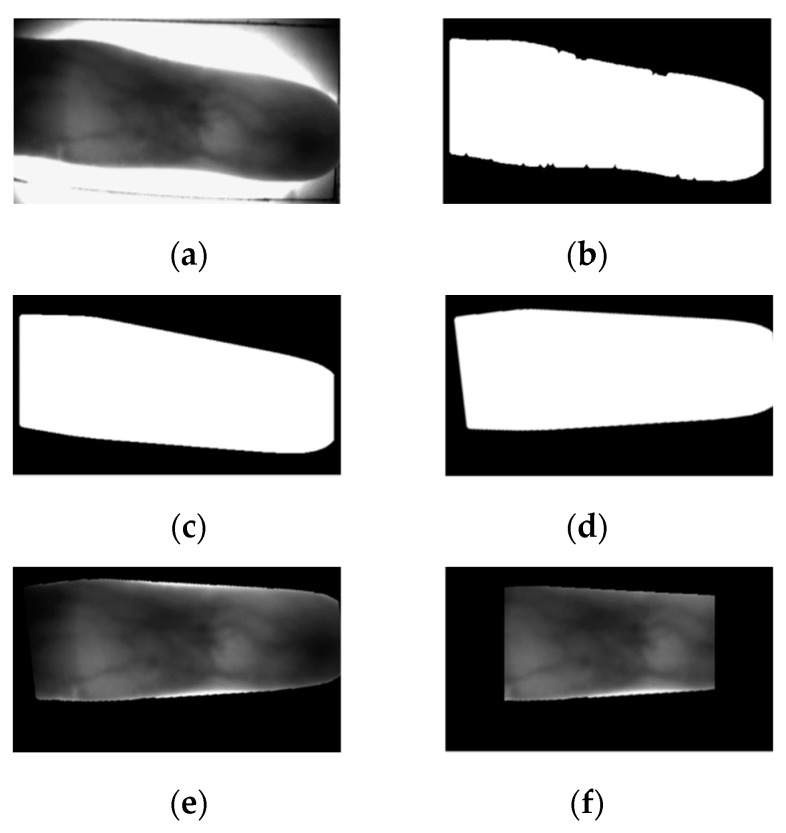
Sample images of each preprocessing stage: (**a**) original image, (**b**) image obtained after Sobel edge detection and thresholding, (**c**) image after edge smoothing, (**d**) image after in-plane rotation compensation, (**e**) finger-vein image obtained by region of interest (ROI) mask, and (**f**) finally cropped finger-vein ROI image.

The obtained finger image has both a background and finger region; therefore, the finger region and the background region need to be primarily segmented to obtain only the finger region in the preprocessing step. Figure 2 shows each preprocessing stage. Binary thresholding and segmentation are performed using the Sobel edge detector and Otsu thresholding method [27]. The image for which binary thresholding has been performed becomes a masked image filled with 255 in the finger region and with 0 for other regions. If the background region and both edges of the finger region have areas with a small pixel value, areas can be mis-classified as the finger region. To remove such areas, both edges are removed and the image is corrected again with component labeling. The boundary of this mask has numerous burrs; thus, a smoothing process would be required to perform accurate linear stretching. Then, in-plane rotation compensation is performed to ensure that the angles of all data are identical. Misalignment in the input image is a major factor that causes false rejection in particular and thus needs to be removed. In-plane rotation compensation involves calculating the second-order angle moments of the binarized mask as shown in Equations (1)–(4), thereby performing misalignment compensation so that all images can have the same angle with respect to the central axis [28].

(1) $$\emptyset_{11}=\frac{\sum_{\left(a,b\right)\in M}{\left(b-m_{b}\right)}^{2}I\left(a,b\right)}{\sum_{\left(a,b\right)\in M}I\left(a,b\right)},$$

(2) $$\emptyset_{22}=\frac{\sum_{\left(a,b\right)\in M}{\left(a-m_{a}\right)}^{2}I\left(a,b\right)}{\sum_{\left(a,b\right)\in M}I\left(a,b\right)},$$

(3) $$\emptyset_{12}=\frac{\sum_{\left(a,b\right)\in M}\left(b-m_{b}\right)\left(a-m_{a}\right)I\left(a,b\right)}{\sum_{\left(a,b\right)\in M}I\left(a,b\right)},$$

(4) $$\tau =\left(\begin{array}{l} arctan\left(\frac{\emptyset_{11}-\emptyset_{22}+\sqrt{{\left(\emptyset_{11}-\emptyset_{22}\right)}^{2}+4\emptyset_{12}{}^{2}}}{-2\emptyset_{12}}\right)\;(if\;\emptyset_{11}\;>\;\emptyset_{22})\\ arctan\left(\frac{-2\emptyset_{12}}{\emptyset_{22}-\emptyset_{11}+\sqrt{{\left(\emptyset_{22}-\emptyset_{11}\right)}^{2}+4\emptyset_{12}^{2}}}\right)\;\left(otherwise\right) \end{array}\right),$$

where I(a,b) and $\left(m_{a},m_{b}\right)$ represent the pixel value and center index in the (a,b) index of the input, M(a,b) represents the pixel value of the mask obtained through binary segmentation; its value should be 255 for the actual finger region and 0 for all other regions. ∅ is the second-order moments for each axis based on which the rotation compensation angle τ is calculated. In detail, $\emptyset_{11}$ and $\emptyset_{22}$ represent the correlation values in the vertical and horizontal directions, respectively. In addition, $\emptyset_{12}$ shows that in the diagonal direction. For example, if $\emptyset_{11}$ is larger than $\emptyset_{22}$, the correlation value of input (I(a,b)) with mask (M(a,b)) in the vertical direction is larger than that in the horizontal direction, which indicates that the input (I(a,b)) with mask (M(a,b)) has the elliptical shape, which is longer in the vertical direction than the horizontal direction. If $\emptyset_{12}$ is larger than $\emptyset_{11}$ and $\emptyset_{22}$, the correlation value of input (I(a,b)) with mask (M(a,b)) in the diagonal direction is larger than those in the vertical and horizontal directions, which indicates that the input (I(a,b)) with mask (M(a,b)) has the elliptical shape, which is longer in the diagonal direction than the vertical and horizontal directions. Based on this information, the rotation compensation angle τ is calculated by Equation (4) [28]. With respect to the central axis, in-plane rotation is performed for the initial finger image and binary mask based on this rotation compensation angle; then, the final finger-vein region is obtained by taking the mask as a condition. In the obtained finger region, the areas in which a finger-vein region cannot be observed easily due to the thickness of the finger or areas in which finger-vein information has been removed due to shades created by the fingernail or bone need to be removed. Therefore, removing a certain portion in the left and right sides of the mask used for acquiring the finger-vein region presents confident finger-vein information.

Certain areas in the mask region, such as the background region represented as a dark area, may be mis-segmented as the finger region during binary thresholding; such areas need to be removed by component labeling [27]. Moreover, if there are areas eroded by additional noise in the finger-shape area, the final ROI mask is obtained through compensation during the smoothing process for removing such areas [26]. The finger region obtained thus undergoes linear interpolation to a size of 256 × 256 to be used as an input of the CycleGAN, which is detailed in the next section.

### 4.3. Domain Adaptation

The existing finger-vein recognition systems are specialized for training data to simply improve performance. However, a finger-vein recognition system is generally used for security purposes; therefore, performance improvement for unobserved data needs to be prioritized. If the image characteristics including brightness, shape, and texture between datasets are different, the network trained with a specific dataset experiences serious performance deterioration when tested with a different dataset. This problem implies that the model lacks generality, and its performance will fluctuate when it is applied in the real world, thus inhibiting the construction of a stable security system. In this study, therefore, both performance and generality are guaranteed by improving the generality in the distribution of the fundamental data through domain adaptation. The network used for domain adaptation in the proposed finger-vein system for this purpose is a CycleGAN.

#### 4.3.1. CycleGAN Architecture

When performing domain adaptation for finger-vein images, there is a high possibility that the features generated in a latent space cannot encompass all the data distribution of each domain if the shape information of the finger-vein is transformed to a high extent. Thus, the image should be generated in a form such that texture information can be inserted while maintaining a shape information of specific domain.

A generative adversarial network which exploits unpaired data is most appropriate for this study because finger-vein image datasets have a different number of classes and thus require unpaired data to be utilized. The purpose is to find the latent space of a new domain between each domain. A CycleGAN uses unpaired data as the source and target; therefore, it can perform a task where the information of the source domain data is retained to some extent while reflecting the target domain information, instead of carrying out a task for simply making the source and target identical [24]. Therefore, a CycleGAN is most appropriate considering these circumstances. A CycleGAN is a network consisting of two discriminators and two generators. 

A 70 × 70 PatchGAN [23] was used as the discriminator. Unlike a general discriminator, PatchGAN is a classifier that discriminates images at a patch unit. The prediction made by a discriminator of a typical GAN is output in an image unit, whereas the prediction made by a discriminator of a PatchGAN is output in a specific patch unit. In other words, the chronic problem of a GAN where blurry output is generated occurs less frequently by determining whether a specific patch region is fake or real, and the process is faster. When the finger-vein shape information used for recognition becomes blurry, the gradient between the finger-vein boundary and skin region is reduced, which implies that it cannot be used effectively. Accordingly, a CycleGAN was selected for domain adaptation in this study.

Table 1 shows the architecture of a 70 × 70 PatchGAN based discriminator. The fake image and original image created in the generator are concatenated to be input. Because it uses a 70 × 70 PatchGAN based method, it is parameter efficient and the relationship between adjacent pixels can be clearly identified based on a local-level discrimination rather than by determining real or fake data in the entire image.

**Figure 3:**
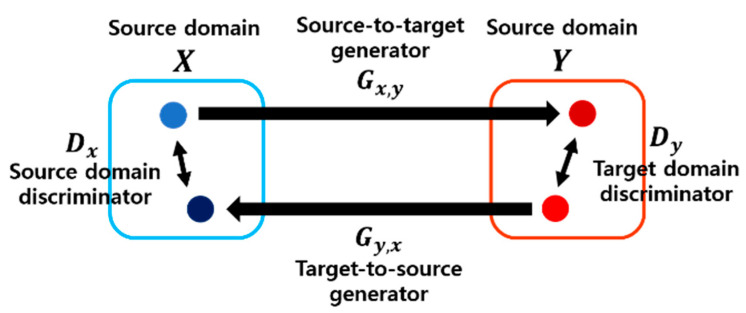
Summary of the CycleGAN structure.

For the generator, a residual network (ResNet) based on an encoder-decoder structural network was used. Figure 3 shows the overall structure of the CycleGAN. Table 2 presents the detailed network architecture of the generator. We use the same settings of parameters and number of layers to those of [24] in Table 1 and Table 2.

#### 4.3.2. Generating a Domain Adapted Finger-Vein Image

**Figure 4:**
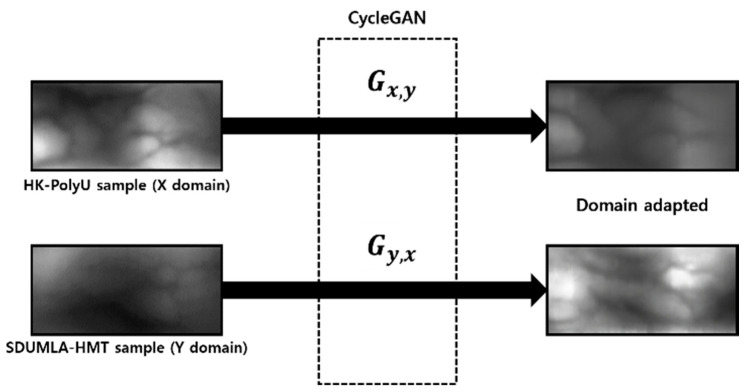
Examples of the domain translated image.

The data of each domain are used as a source and a target of the CycleGAN to generate an image for which domain adaptation has been applied. Figure 4 shows an example of the domain adapted image. It resembles the shape of an image used as a source and shows the shape in which the distribution of lighting intensity or contrast of the target domain is reflected. Hence, an image of a new domain is obtained for which information is composited.

The loss function of a CycleGAN is the weighted sum of adversarial loss and cycle-consistency loss (see Equations (7)–(9)). The purpose of a generator is to deceive a discriminator by generating fake data that resemble the real data as much as possible, whereas a discriminator is trained to distinguish fake data from real data. Comparing the real data and simply generated data generates adversarial loss, as shown in Equations (5)–(7), while cycle-consistency loss helps in building a robust model through reconstruction by comparing the real data with source data, as shown in Equation (8). Ultimately, the loss function in which both adversarial loss and cycle-consistency loss are considered, as shown in Equation (9), is used. We use the same loss functions of Equations (5)–(9) to those in traditional CycleGAN [24].

(5) $$Loss_{adv}\left(G_{x,y},D_{y},\;X\right)=\;\frac{1}{m}\overset{m}{\underset{i=1}{\sum }}{\left(1-D_{y}\left(G_{x,y}\left(x_{i}\right)\right)\right)}^{2},$$

(6) $$Loss_{adv}\left(G_{y,x},D_{x},\;Y\right)=\;\frac{1}{m}\overset{m}{\underset{i=1}{\sum }}{\left(1-D_{x}\left(G_{y,x}\left(y_{i}\right)\right)\right)}^{2},$$

(7) $$Loss_{adv}=Loss_{adv}\left(G_{x,y},D_{y},\;X\right)+Loss_{adv}\left(G_{y,x},D_{x},\;Y\right),$$

(8) $$Loss_{cyc}\left(G_{x,y},G_{y,x},X,\;Y\right)=\frac{1}{m}\overset{m}{\underset{i=1}{\sum }}\left(\left(G_{x,y}\left(G_{y,x}\left(y_{i}\right)\right)\right)-y_{i}\right)-\left(\left(G_{y,x}\left(G_{x,y}\left(x_{i}\right)\right)\right)-x_{i}\right),$$

(9) $$Loss_{total}=\;Loss_{adv}+\;\lambda Loss_{cyc},$$

where *G* and *D* represent the generator and discriminator, respectively, $x_{i}$ and $y_{i}$ are the source image and target image selected in the X, Y domain, respectively, and m is the total number of data of each domain. λ is a cycle-consistency coefficient; a value of 10 was used in this study. Processing heterogeneous data through domain adaptation, as proposed in this study, enables us to retain the shape information of a specific domain while generating new domain data through adaptation of the texture information of a different domain. Thus, for a proper mixture of shape information and texture information, cycle-consistency loss value and adversarial loss value were adjusted using λ. 

### 4.4. Generating Composite Image

**Figure 5:**
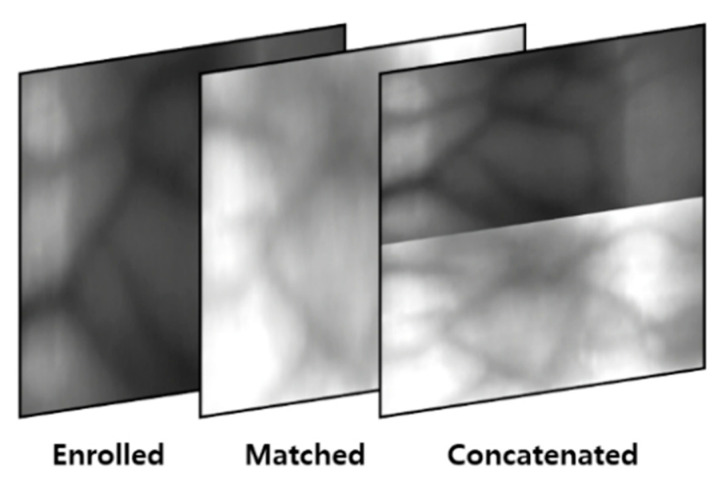
Example of composite image.

A composite image is generated using the domain adapted image [26]. It is generated for a matching case, and it maximizes the network utilization rate more than the feature-based Euclidean distance matching method used in conventional finger-vein recognition systems. For the feature-based Euclidean distance matching method, matching is performed using the features extracted before the fully connected layer in a trained CNN model for the finger-vein recognition system. Thus, a trained fully connected layer cannot be used. In contrast, when generating authentic and imposter matching images as composite images, all layers in the trained CNN model for finger-vein recognition including the fully connected layer can be used. Furthermore, a data augmentation effect is observed during training because composite images are generated for the number of matching cases, and it is more robust for noise than difference image-based matching [5]. As shown in Figure 5, a composite image is an image generated by having an enrolled image, a matched image, and a concatenated image in each channel. The concatenated image is created by resizing the enrolled image and the matched image into 1/2 size images and then concatenating vertically. As a result, a three-channel shape image is generated and input in the CNN classifier. The composite image-based method does not involve Euclidean distance calculation by a n-dimensional feature vector, thus requiring a shorter time during inference compared to feature distance-based matching.

### 4.5. Finger-Vein Recognition Based on Deep Densenet and Shift Matching

In this study, DenseNet-161 was used as the model for finger-vein recognition [26,29]. Table 3 represents architecture of DenseNet-161 that used in this study. We use the same settings of parameters and number of layers to those of [29] in Table 3. In the DenseNet-161 used for proposed method, the growth rate was set to 48. The original structure of DenseNet was designed for ImageNet classification [29]. The output of the fully connected layer was a 1000-dimensional vector. As only two types of output—authentic matching score and imposter matching score—are used in this study, the existing fully connected layer was removed and fine tuning was performed after replacing it with a fully connected layer that outputs a two-dimensional score vector. DenseNet can effectively convey low level features to deeper layers through a dense connection.

Therefore, DenseNet was determined to be a very suitable classifier because low level features such as a ridge are the core components of the vein shape information present in the finger-vein data used in this study. For the composite image generated by acquiring the domain adapted image, the enrolled image and matched image are input in the same DenseNet-161. The spatial similarity of each image was evaluated in the classifier to confirm whether it is an authentic matching case or an imposter matching case. However, while evaluating the spatial similarity, misalignment or rotation, which were not removed during preprocessing, could be observed. These factors significantly affect the process of matching. To solve these problems, the enrolled image or matched image was matched through eight-way translation in this study. Then, the misalignment issue such as pixel translation was solved by designating the minimal matching value as the final matching score.

## 5. Experimental Results

In this section, we would explain experimental environments in Section 5.1, training of the domain adaptation model in Section 5.2, and training of finger-vein recognition model in Section 5.3. In addition, we would explain evaluation metrics in Section 5.4, and testing results and analyses with HKPolyU-DB after training with SDUMLA-HMT-DB (including ablation study) in Section 5.5. Finally, testing results and analyses with SDUMLA-HMT-DB after training with HKPolyU-DB (including ablation study) are presented in Section 5.6.

### 5.1. Experimental Environments

In this study, SDUMLA-HMT-DB [30] and HKPolyU-DB version 1 [31] were used. The HKPolyU database is divided into session 1 and session 2; only session 1 data were used in this study. HKPolyU-DB session 1 consists of 1872 images; two fingers of 156 persons were used for image acquisition, and six images were captured for each finger. SDUMLA-HMT-DB consists of 3816 images in which three fingers of each hand of 106 persons were used, and six images were captured for each finger. Each dataset was classified according to the finger used to acquire the image. HKPolyU-DB and SDUMLA-HMT-DB have a total of 312 classes and 636 classes, respectively. The number of classes is calculated by “the number of fingers” × “the number of hands” × “the number of persons”. For example, because “the number of fingers”, “the number of hands”, and “the number of persons” in SDUMLA-HMT-DB are 3, 2, and 106, respectively, the number of classes becomes 636 (3 × 2 × 106) in SDUMLA-HMT-DB. To perform two-fold cross validation for training and testing, 156 classes were used for the training set and another 156 classes were used for the testing set for HKPolyU-DB, whereas 318 classes were used for the training set and another 318 classes were used for the testing set for SDUMLA-HMT-DB in 1st-fold validation. Specifically, the training and testing datasets did not include data from the same class. The training set and testing set were switched once for the experiment in the second-fold validation, and the average of the two accuracy values was used as the final value. In detail, as shown in Table 4, in the first-fold validation, the images of 318 classes (classes 1~318) were used for training whereas those of the remaining 318 classes (classes 319~636) were used for testing. In the second-fold validation, the images of 318 classes (classes 319~636) were used for training whereas those of the remaining 318 classes (classes 1~318) were used for testing. The sets used in each database are summarized in Table 4.

**Figure 6:**
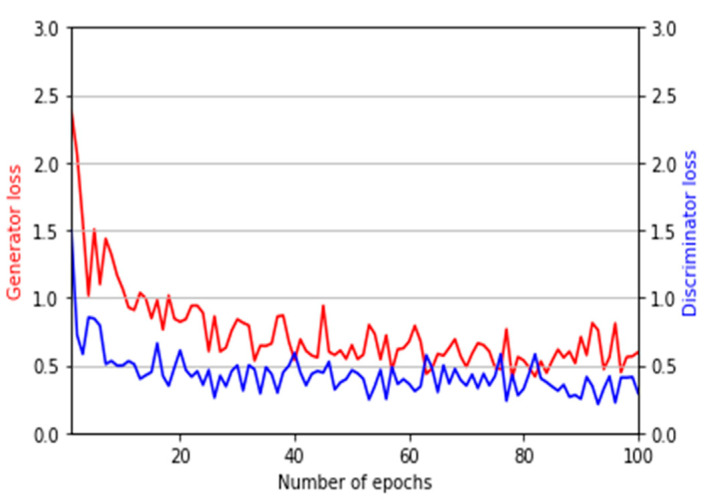
Graphs of training loss and accuracy by CycleGAN.

**Figure 7:**
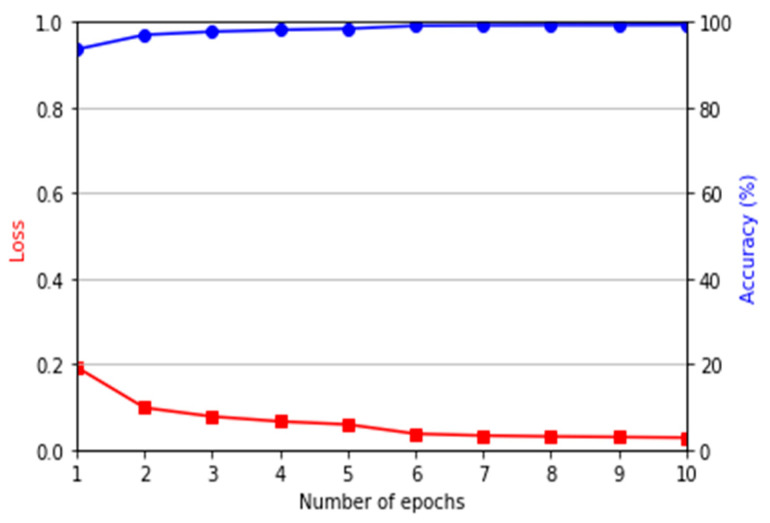
Graphs of the training loss and accuracy by DenseNet-161 using domain adapted data.

We increased the number of training images by five times (including original training images) based on the data augmentation of image translation and cropping in the four directions (left, right, up, and down directions) by referring to [4]. Therefore, the total number of training images in HKPolyU-DB is 4680 (936 × 5) for each fold, and that in SDUMLA-HMT-DB is 9540 (1908 × 5) for each fold as shown in Table 4. With these augmented data, our models for domain adaptation and finger-vein recognition were successfully trained as shown in Figure 6 and Figure 7.

When we generated the images from HKPolyU-DB by CycleGAN, the test images of HKPolyU-DB were used for generation. Therefore, the number of generated images is 936 as shown in Table 4. When we generated the images from SDUMLA-HMT-DB by CycleGAN, the test images of SDUMLA-HMT-DB were used for generation. Therefore, the number of generated images is 1908 as shown in Table 4.

Training and testing were performed using a desktop computer equipped with an Intel^®^ Core™ i7-3770K CPU @ 3.50GHz with 12GB RAM, and the graphics processing unit (GPU) card of NVIDIA Geforce GTX 1070 [32]. Moreover, compute unified device architecture (CUDA) version 9.0 [33] and CUDA deep neural network library (CUDNN) version 7.4.2 [34] were used. To execute the model and algorithm proposed in this study, Tensorflow framework version 1.15.1 [35] based on Python version 3.7.1 [36] was used.

### 5.2. Training of the Domain Adaptation Model

For the optimizer of the CycleGAN used for domain adaptation, the adaptive moment estimation (Adam) optimizer [37] was used. The initial learning rate was 0.0001; the exponential decay rate of the Adam optimizer was 0.9 for the first moment estimate and 0.999 for the second moment estimate. The learning rate strategies such as linear decay were not used. The model was trained for a total of 100 epochs. The discriminator was trained once for one mini-batch, whereas the generator was trained five times to solve the problem of the difficulty in training the generator of CycleGAN. Owing to this training strategy, the CycleGAN model used in this study was appropriately optimized for both the discriminator and generator. Figure 6 shows the loss graph of the generator and discriminator of the CycleGAN used in this study.

### 5.3. Training of Finger-Vein Recognition Model

A transfer learning strategy was used for training the finger-vein recognition model. The fully connected layer of the original network fine-tuned with the ImageNet was replaced with a fully connected layer with two-dimensional output, thus freezing the previous convolutional layer part and using the fully connected layer in the domain adapted image for training. Figure 7 shows the loss and accuracy graphs of the DenseNet-161 used in this study. These graphs imply that the DenseNet-161 model has been appropriately optimized.

### 5.4. Evaluation Metrics

An EER was used as the evaluation metric in this experiment. Each input determines genuine matching cases and imposter matching cases based on the matching score obtained during finger-vein recognition. Here, the rate of cases in which imposter matching cases have been categorized as genuine matching cases is the false acceptance rate (FAR), whereas the rate of cases in which the genuine matching cases are categorized as the imposter matching cases is the false rejection rate (FRR). The final EER is obtained at the threshold point where FAR and FRR are the same.

### 5.5. Testing with HKPolyU-DB after Training with SDUMLA-HMT-DB (including Ablation Study)

In this section, the results of the experiment which proved the effect of the database that has been domain adapted from HKPolyU-DB to SDUMLA-HMT-DB are presented. As shown in Table 5, our CycleGAN was trained with the training data of HKPolyU-DB (input domain) and SDUMLA-HMT-DB (target domain), and the trained CycleGAN generated the domain adapted image (similar to the images of SDUMLA-HMT-DB) by using the testing data of HKPolyU-DB. Then, for testing, the generated images (similar to the images of SDUMLA-HMT-DB) were used as input to our finger-vein recognition model trained with the training data of SDUMLA-HMT-DB.

For two-fold cross validation, the model for domain adaptation was trained using the training set. When both types of databases (HKPolyU-DB, SDUMLA-HMT-DB) were used during domain adaptation, the training set and the testing set were strictly separated for both databases in two-folds. Accordingly, the experiment was performed in an open-world setting in which the class of training data was different from the class of testing data.

Table 6 shows the comparison of the drop of finger-vein recognition performance for the same domain and cross-domain environment while the DenseNet-161 network is applied in the same manner without the CycleGAN-based domain adaptation proposed in this study.

**Figure 8:**
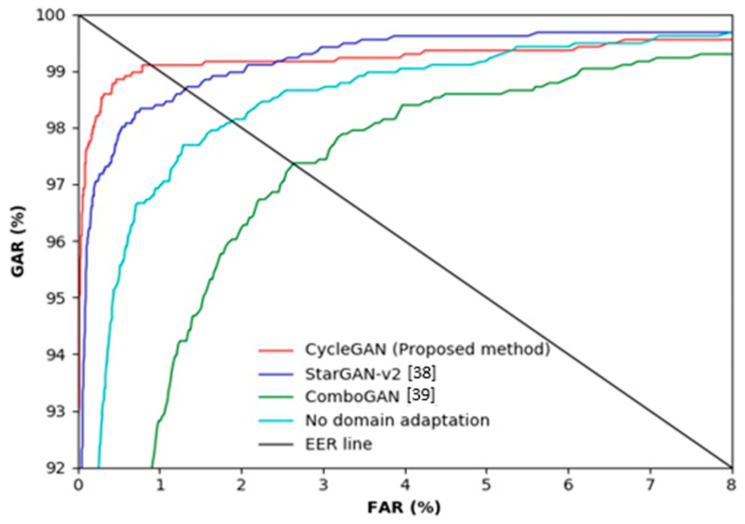
ROC curves of finger-vein recognition by the proposed method and other methods in case of training with the SDUMLA-HMT database and testing with the HKPolyU database.

**Figure 9:**
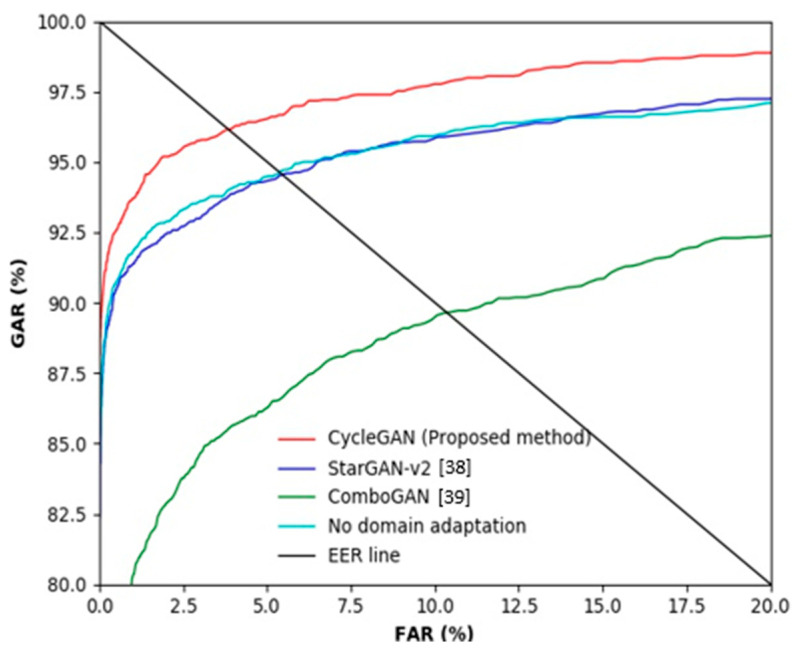
ROC curves of finger-vein recognition by the proposed method and other methods in case of training with HKPolyU-DB and testing with SDUMLA-HMT-DB.

As shown in Table 6, when training and testing were conducted using HKPolyU-DB, the recognition rate was high with the EER of 0.58%. In contrast, when the model was trained using SDUMLA-HMT-DB and tested using HKPolyU-DB without the CycleGAN-based domain adaptation, the accuracy was significantly lower. As shown in Table 4, the amount of data used in SDUMLA-HMT-DB were considerably greater than that used on in HKPolyU-DB, and performance drop occurred even though they are both databases of the same finger-vein scope. The difference in data between the two domains is not visually noticeable; however, the heterogeneity between the two domains is definitely present. Moreover, the qualities of images in HKPolyU-DB are relatively better than those of images in SDUMLA-HMT-DB, and the intra-class variance is lower. In other words, the training set is a much more complex case than the testing set; thus, the performance drop is not significant. However, compared with the same domain environment, the cross-validation environment experienced a considerable performance drop, and the domain adaptation method was used to solve this problem. Table 7 and Figure 8 show the accuracy of finger-vein recognition of the various domain adaptation methods. Here, genuine acceptance rate (GAR) is defined as 100–FRR (%). Therefore, we can find that the ratio of FRR to FAR is smaller in case that the ROC curve is positioned higher (closed to the left-top position of Figure 8 and Figure 9), which means the ratio of GAR to FAR is higher. The experimental results showed that the accuracy is significantly higher when the proposed CycleGAN-based method is used compared to the cases when a domain adaptation method is not applied or other domain adaptation methods were used. This result implies that domain adaptation based on the proposed method sufficiently transferred the feature information of each domain.

Table 8 shows a comparison of the accuracy of the proposed method and the state-of-the-art methods. The experimental results highlighted that the proposed method had a higher recognition accuracy than the state-of-the-art methods.

### 5.6. Testing with SDUMLA-HMT-DB after Training with HKPolyU-DB (including Ablation Study)

In this section, we performed the experiments again by exchanging HKPolyU-DB and SDUMLA-HMT-DB compared to the experiments of Section 5.5. Table 9 shows the result of performing training and testing with SDUMLA-HMT-DB and of performing training with HKPolyU-DB and testing with SDUMLA-HMT-DB. The performance drop is greater compared to the result shown in Table 6, which can be because the degree of noise, misalignment, and blur in the images in SDUMLA-HMT-DB are considerably greater than those of the images in HKPolyU-DB. Therefore, the recognition performance in the cross-domain environment is significantly low because of the unique trait of the domain transformed by noise or an image capturing device.

Table 10 and Figure 9 show the accuracy of finger-vein recognition obtained by various domain adaptation methods. The experimental results showed that the accuracy is significantly higher when the proposed CycleGAN-based method is used compared to when a domain adaptation method is not applied or other domain adaptation methods were used. Thus, the feature information that can be obtained from SDUMLA-HMT-DB has been well adapted while partially maintaining the unique shape information of HKPolyU-DB. The results of StarGAN-v2 and ComboGAN are poorer than those of the proposed CycleGAN. Table 7 and Figure 8 present similar results. Fundamentally, a CycleGAN is a network designed for style transfer between two domains, whereas ComboGAN and StarGAN-v2 are designed for multi-domain transfer. Particularly, a StarGAN-v2 can not only simply discriminate between real or fake data using a style code but can also discriminate the type of domain generated. In a multi-domain focused architecture, performance is poorer as the discrepancy between domains is greater. Only a specific region cannot have high activation due to the trait of finger-vein data, and the heterogeneity in the shape information is noticeably significant even if the databases appear similar. Furthermore, ComboGAN not only mitigates the number of generators which increases with multi-domain transfer cases but also attempts to solve the problem of deteriorating performance caused by a greater difference in the domains of the existing StarGAN. However, the encoder and decoder separated by the number of domains recognize a specific database as one style as proposed by the ComboGAN, i.e., it failed to completely learn the domain distribution.

Table 11 shows the comparison of the accuracy between the proposed method and the state-of-the-art methods. The experimental result showed that the proposed method had a higher recognition accuracy than the state-of-the-art methods.

**Figure 10:**
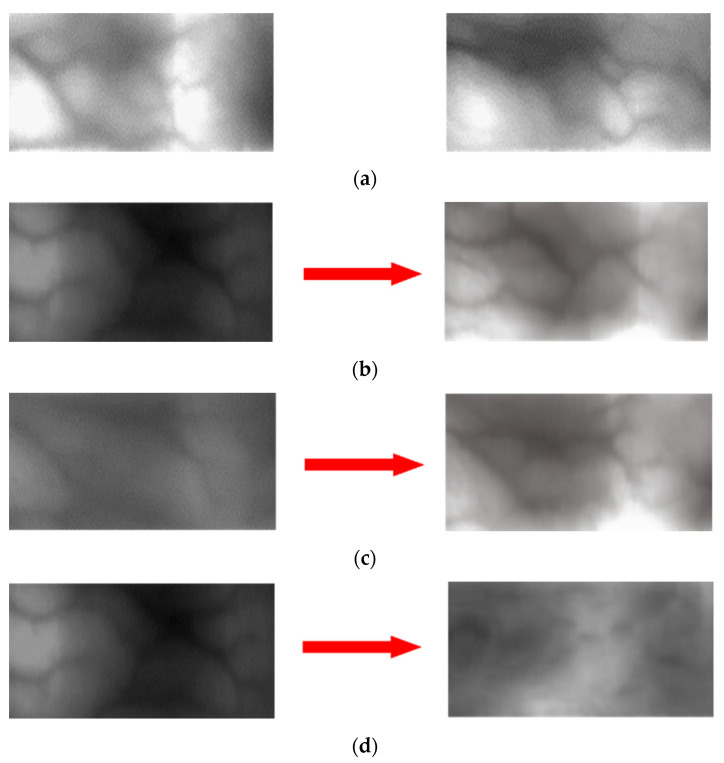

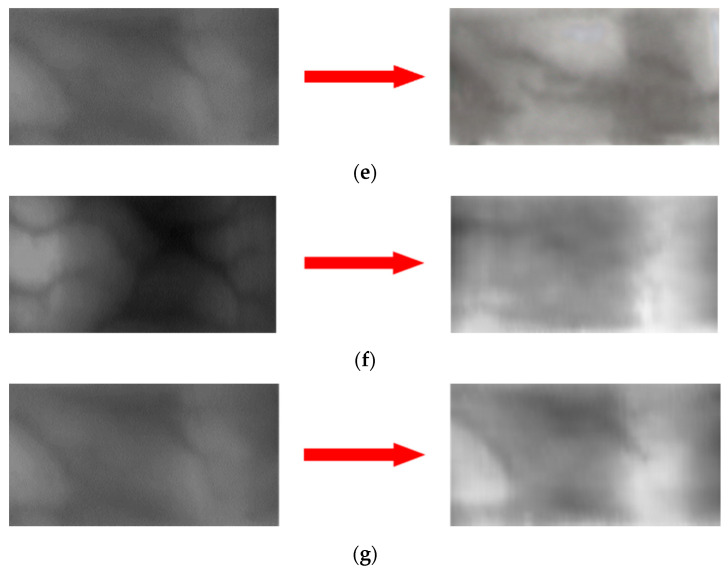
Examples of original images and domain adapted images: (**a**) Original image of HKPolyU-DB. Left and right images of (**b**–**g**) respectively, show original images and domain adapted images from SDUMLA-HMT-DB and HKPolyU-DB using various methods. (**b**,**c**) proposed method, (**d**,**e**) ComboGAN, (**f**,**g**) StarGAN-v2.

**Figure 11:**
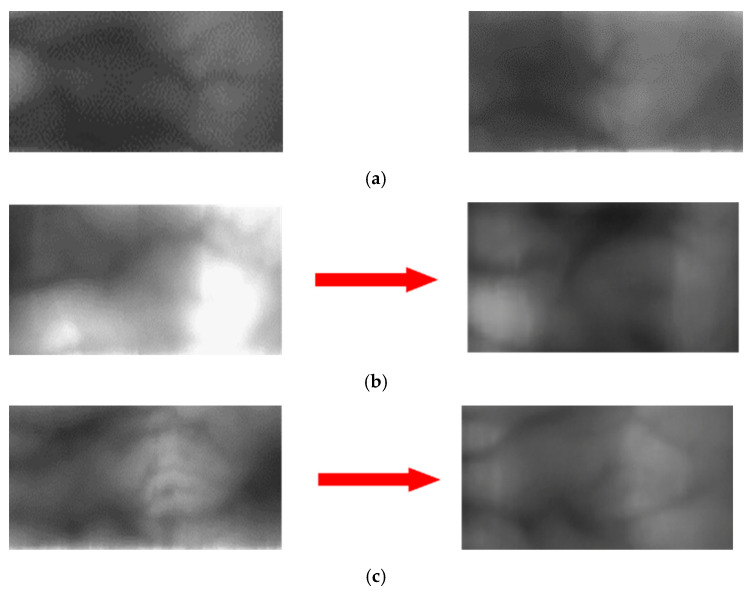

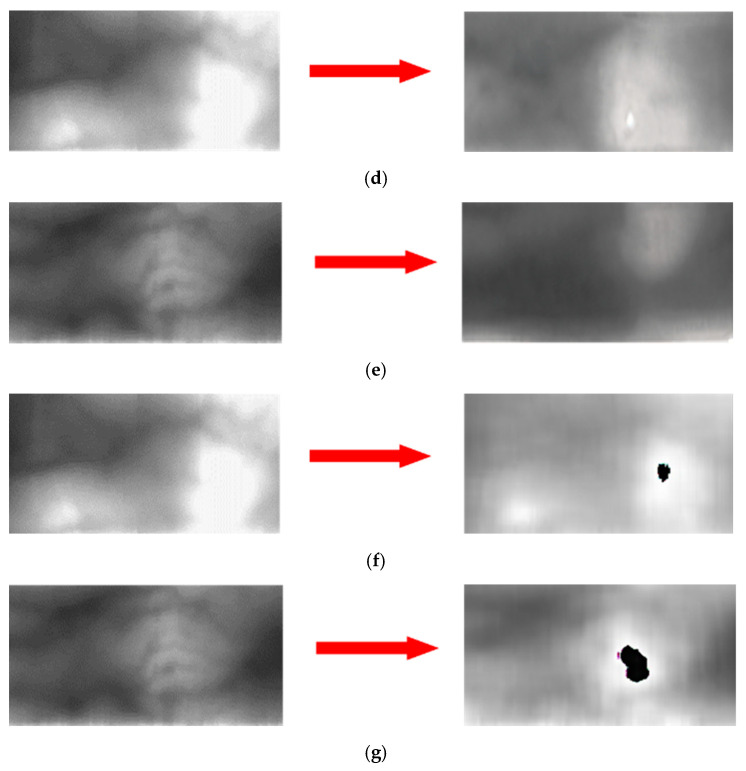
Examples of original images and domain adapted images: (**a**) Original image of SDUMLA-HMT-DB. Left and right images of (**b**–**g**), respectively, show original images and domain adapted images from HKPolyU-DB to SDUMLA-HMT-DB using various methods. (**b**,**c**) proposed method, (**d**,**e**) ComboGAN, (**f**,**g**) StarGAN-v2.

Figure 10 and Figure 11 show examples of the image domains adapted using various methods. Figure 10a and Figure 11a show the examples of the original image; the images on the left in (b)–(g) are the source images and those on the right are images generated through domain adaptation using the source images. That is, the left and right images of Figure 10b–g, respectively, show original images and domain adapted images from SDUMLA-HMT-DB and HKPolyU-DB using various methods ((b), (c) our method, (d), (e) ComboGAN, (f), (g) StarGAN-v2). By comparing the right images of (b) and (c) with those of (d)–(g), the right images of (b) and (c) by our method have more similar image characteristics (including the distinctiveness of vein patterns) to the original images of HKPolyU-DB (Figure 10a) compared to the right images of (d)–(g). In addition, as shown in Figure 11, by comparing the right images of (b) and (c) by our method with those of (d)–(g) by other methods, the right images of (b) and (c) have more similar image characteristics (including to the distinctiveness of vein patterns) to the original images of SDUMLA-HMT-DB (Figure 11a) compared to the right images of (d)–(g). 

As shown in all examples, the image generated by the proposed method using a CycleGAN has the best quality; the images generated by the StarGAN-v2 are somewhat blurry and exhibit dark noises while transferring the target domain style to a certain extent. Lastly, the image generated by the ComboGAN shows that the difference in data quantity between SDUMLA-HMT-DB and HKPolyU-DB as well as the separated encoder and decoder structure did not produce good performance. Unlike facial emotion data in which features are concentrated in specific regions, the information is not concentrated in specific regions in the finger-vein data; thus, it is difficult to assign a style. Therefore, the results in Figure 10 and Figure 11 are produced if the generator structure is not concrete because the dataset is widely distributed.

**Figure 12:**
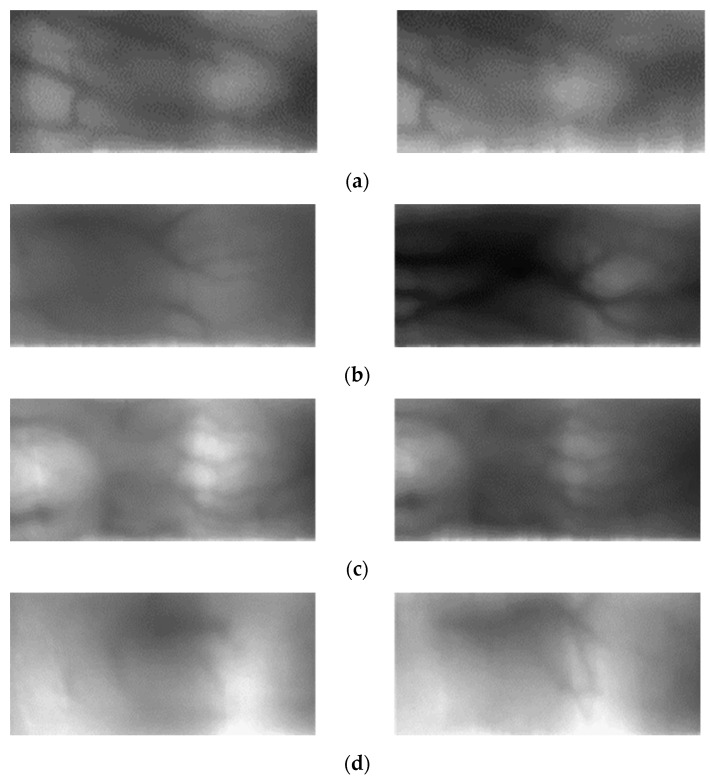
Examples of errors in case of testing with HKPolyU-DB: (**a**) False rejection case by both proposed method and no domain adaptation, (**b**) false acceptance case by both proposed method and no domain adaptation, (**c**) false rejection case by the no adaptation method, but correct recognition case by the proposed method, (**d**) false acceptance case by the no adaptation method, but correct rejection case by the proposed method. Left and right images of (**a**–**d**) show enrolled and matched images, respectively.

Finally, the effect of the proposed method was analyzed by comparing the cases in which recognition errors were produced in all schemes in which the proposed method and domain adaptation were not applied and cases in which the model correctly recognized the images only using the proposed method. Figure 12 summarizes the error cases generated in the no adaptation method where SDUMLA-HMT-DB was used as the training set and in the proposed method where SDUMLA-HMT-DB was domain adapted to HKPolyU-DB. Figure 12a,b show the cases in which errors occurred even when domain adaptation was performed using the proposed method. Specifically, Figure 12a is an example of a false rejection case, and Figure 12b is the example of a false acceptance case. As shown in Figure 12a, a major pixel translation observed even when the enrolled image and the matched image were an authentic matching case. In Figure 12b, both images were not properly acquired because of the imbalance in lighting intensity of the NIR sensor used for acquiring the finger-vein images. Because of these problems, the finger-vein pattern appeared only in a limited region of the image, which resulted in an imposter matching case which appeared as an authentic matching case. In addition, correctly recognizing if the shape pattern, which is important information, is distributed in a similar manner, is a challenging task.

Figure 12c,d are the results of correct recognition when the proposed method was used in which 12c shows the falsely rejected case and 12d shows the falsely accepted case in a scheme where the domain adaptation method was not applied. Figure 12c is an authentic matching case; however, a problem was observed when the intensity of lighting varied during the image capturing trial. However, the data for which domain adaptation was performed are effective against the variance in lighting intensity as such information of the source domain, SDUMLA-HMT-DB, was also transferred. Figure 12d also shows that it is difficult to identify the overall finger-vein pattern because finger-vein information is acquired from a limited region; however, a good recognition performance was still observed when the proposed method was used appropriately using the scarcely available finger-vein pattern. Therefore, a robust performance was achieved for extracting the finger-vein valley through domain adaptation.

**Figure 13:**
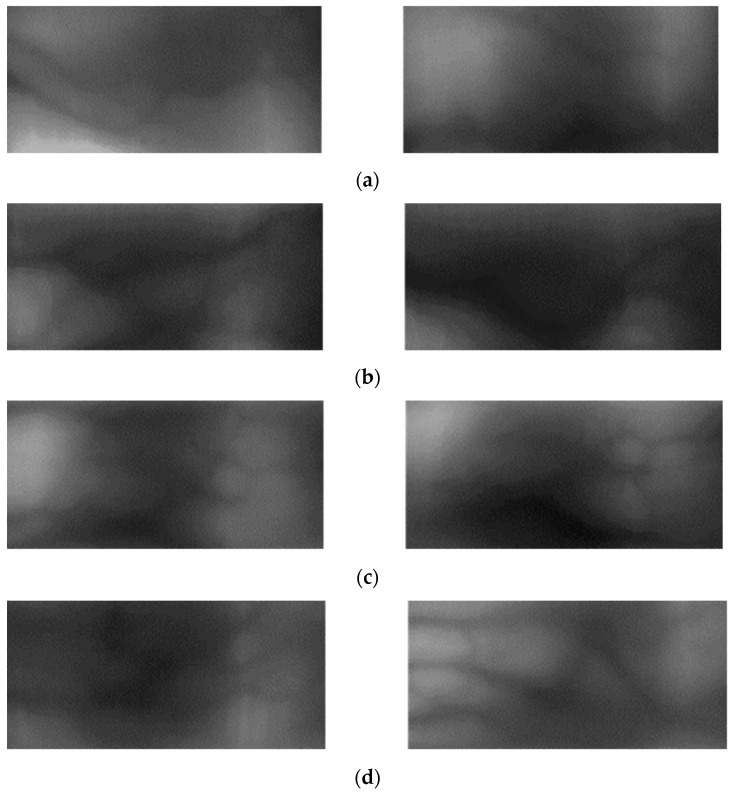
Examples of errors in the case of testing with SDUMLA-HMT-DB: (**a**) False rejection case by both the proposed method and no domain adaptation, (**b**) false acceptance case by both the proposed method and no domain adaptation, (**c**) false rejection case by the no adaptation method, but correct recognition case by the proposed method, (**d**) false acceptance case by the no adaptation method, but correct rejection case by the proposed method. Left and right images of (**a**–**d**) show enrolled and matched images, respectively.

Unlike Figure 12, Figure 13 summarizes the error cases generated in the no adaptation method where HKPolyU-DB was used as the training set, and in the proposed method where HKPolyU-DB was domain adapted to SDUMLA-HMT-DB. The information was mostly not contained in the images properly for the data of SDUMLA-HMT-DB, which is similar to the data of HKPolyU-DB. In particular, the cases in Figure 13a,b only contained a small amount of finger-vein patterns, and the recognition was performed using the background information during testing. This problem cannot be easily solved by domain adaptation, and therefore, it was not successfully recognized in the case where the proposed method was used. Even though the case in Figure 13c is an authentic matching case, the pixel translation between the enrolled image and the matched image was significantly large, while the forms of the shades slightly varied. However, for the data generated using the proposed method, the finger-vein pattern of each domain was effectively transferred, thus producing robust performance for the finger-vein pattern of SDUMLA-HMT-DB along with the focused form of the finger-vein pattern. This shows that the network was optimized to generate variations in the vein pattern information by focusing on the vein pattern when training the CycleGAN. Figure 13d also shows that it is difficult to identify the overall finger-vein pattern because the finger-vein information is acquired from a limited region; however, a good recognition performance was still observed when the proposed method was used appropriately using the scarcely available finger-vein pattern.

## 6. Discussion

In this section, we briefly compared the previous and proposed methods with advantages and disadvantages, as shown in Table 12. 

In case of five-fold or 10-fold cross validation, the number of training data becomes much larger, and the consequent accuracy of testing becomes higher than that by two-fold cross validation in most cases due to the sufficient training of model. However, it is very difficult to acquire the sufficient number of training data in real world cases. Considering these cases, we aim at measuring the testing accuracies even with insufficient training data based on two-fold cross validation in our experiments.

The proposed method failed the correct recognition in the following cases; (i) a major pixel translation observed even when the enrolled image and the matched image were an authentic matching case, (ii) both the enrolled and matched images were not properly acquired because of the imbalance in lighting intensity of the NIR sensor used for acquiring the finger-vein images, and (iii) the captured image only contained small amount of finger-vein patterns, and the recognition was performed using the background information during testing.

## 7. Conclusions

In this study, we propose a finger-vein recognition system in which domain adaptation is applied to solve the problem of the performance drop in a finger-vein recognition system when unobserved data are used. Domain adaptation was performed using CycleGAN, and the proposed domain adapted model proved to be effective using two databases—HKPolyU-DB and SDUMLA-HMT-DB. All cases found in the real environment include unobserved data; thus, a performance drop in similar circumstances is critical. As a finger-vein recognition system is used for security purposes, unstable performance depending on specific situations would decrease the reliability, thus making its application to real-world applications difficult. Using the proposed method, a stable finger-vein recognition system with improved generality can be applied to various real-world applications.

In this research, we focused on checking the possibility of domain adaptation of heterogeneous finger-vein databases. Therefore, we used the well-known CycleGAN and DenseNet-161 whose performances for style transfer of unpaired data and classification were already confirmed, respectively, in many previous researches of different applications. We performed only the fine-tuning of CycleGAN and DenseNet-161 with our experimental data. We would research the method of further customization of CycleGAN and DenseNet-161 to enhance the accuracies as future works.

In addition, we would research the advanced domain adaptation method, which can solve the cases of major pixel translation between the enrolled and matched images, the imbalance of lighting intensity in the captured image, and the small amount of finger-vein patterns contained in the captured image explained in Section 6. We would also evaluate the performance by five-fold or 10-fold cross validation in future work. In addition, a finger-vein recognition system with a more robust performance for unobserved data will be further studied in the future by improving the generality of the domain through multiple-domain adaptation, rather than simple domain adaptation between two databases. Furthermore, the efficacy of domain adaptation proposed in this study will also be researched for diverse biometric data such as palm and hand dorsal vein images, visible and NIR iris images, and visible and NIR face images.

## Figures and Tables

**Table 1:** Architecture of the discriminator used in CycleGAN.

Layer	Filter (Number/Size/Stride)	Input Size	Output Size
Input layer		256 × 256 × 3 (×2)	256 × 256 × 6
Conv1 *	64/4 × 4 × 3/2	256 × 256 × 6	128 × 128 × 64
Conv2 *	128/4 × 4 × 64/2	128 × 128 × 64	64 × 64 × 128
Conv3 *	256/4 × 4 × 128/2	64 × 64 × 128	32 × 32 × 256
Conv4 *	512/4 × 4 × 256/1	32 × 32 × 256	31 × 31 × 512
Conv5	1/4 × 4 × 512/1	31 × 31 × 512	30 × 30 × 1

* denotes that the convolutional layer is followed by instance normalization and a leaky rectified linear unit (ReLU) with a slope parameter of 0.2.

**Table 2:** Architecture of the generator used in CycleGAN.

Layer	Filter (Number/Size/Stride)	Input Size	Output Size
Input layer		256 × 256 × 3	256 × 256 × 3
Conv1	64/7 × 7 × 3/1	256 × 256 × 3	256 × 256 × 64
Conv2 *	128/3 × 3 × 64/2	256×256×64	128 × 128 × 128
Conv3 *	256/3 × 3 × 128/2	128 × 128 × 128	64 ×64 × 256
Res1	(256/3 × 3 × 256/1) × 3 **	64 × 64 × 256	64 ×64 × 256
Res2	(256/3 × 3 ×256/1) × 3 **	64 × 64 × 256	64 ×64 × 256
Res3	(256/3 × 3 × 256/1) × 3 **	64 × 64 × 256	64 ×64 × 256
Res4	(256/3 × 3 × 256/1) × 3 **	64 × 64 × 256	64 ×64 × 256
Res5	(256/3 × 3 × 256/1) × 3 **	64 × 64 × 256	64 ×64 × 256
Res6	(256/3 × 3 × 256/1) × 3 **	64 × 64 × 256	64 ×64 × 256
Res7	(256/3 × 3 × 256/1) × 3 **	64 × 64 × 256	64 ×64 × 256
Res8	(256/3 × 3 × 256/1) × 3 **	64 × 64 × 256	64 ×64 × 256
Res9	(256/3 × 3 × 256/1) × 3 **	64 × 64 × 256	64 ×64 × 256
Up-conv1	128/3 × 3 × 256/2	64 × 64 × 256	128 ×128 × 128
Up-conv2	64/3 × 3 × 256/2	128 × 128 × 128	256 × 256 × 64
Conv4	3/7 × 7 × 3/1	256 × 256 × 64	256 × 256 × 3

* denotes that the convolutional layer is followed by instance normalization and ReLU. ** denotes that the Res(k) is a residual block where an input feature map is added to the output of each residual block, and each residual block includes three convolutional layers.

**Table 3:** Architecture of DenseNet-161.

Layer	Filter (Number/Size/Stride)	Input Size	Output Size
Input layer		224 × 224 × 3	224 × 224 × 3
Conv	(96/7 × 7 × 96/2)	224 × 224 × 3	112 × 112 × 96
Max pool	(96/2 × 2 × 1/2)	112 × 112 × 96	57 × 57 × 96
Dense block	(6/(1 × 1 × 192, 3 × 3 × 48)/1)	57 × 57 × 96	57 × 57 × 384
Transition block	(1/(1 × 1 × 192, 2 × 2 × 192) */1)	57 × 57 × 384	29 × 29 × 192
Dense block	(12/(1 × 1 × 192, 3 × 3 × 48)/1)	29 × 29 × 192	29 × 29 × 768
Transition block	(1/(1 × 1 × 384, 2 × 2 × 384) */1)	29 × 29 × 768	15 × 15 × 384
Dense block	(36/(1 × 1 × 192, 3 × 3 × 48)/1)	15 × 15 × 384	15 × 15 × 2112
Transition block	(1/(1 × 1 × 1056, 2 × 2 × 1056) */1)	15 × 15 × 2112	8 × 8 × 1056
Dense block	(24/(1 × 1 × 192, 3 × 3 × 48)/1)	8 × 8 × 1056	8 × 8 × 2208
Global average pool	(2208/8 × 8 × 1/1)	8 × 8 × 2208	1 × 1 × 2208
Fully connected layer		1 × 1 × 2208	1 × 1 × 2

* denotes the shape of the convolutional filter and average pooling filter, respectively.

**Table 4:** Details of the experimental databases.

Database	Subset	Classes	Number of Original Images	Number of Augmented Images
HKPolyU-DB	Training	156	936	4680
	Test	156	936	-
SDUMLA-HMT-DB	Training	318	1908	9540
	Test	318	1908	-

**Table 5:** Experimental scenario of our domain adaptation method (unit: %).

Training of CycleGAN	Image Generation by CycleGAN	Training of Finger-Vein Recognition Model	Testing of Finger-Vein Recognition Model
Using the training data of HKPolyU-DB (input domain) and SDUMLA-HMT-DB (target domain)	Using the testing data of HKPolyU-DB	Using the training data of SDUMLA-HMT-DB	Using the generated images by CycleGAN (similar to SDUMLA-HMT-DB)

**Table 6:** Comparisons of EER with same domain and cross-domain environment without our domain adaptation method (unit: %).

Training of Finger-Vein Recognition Model	Testing of Finger-Vein Recognition Model	EER
HKPolyU-DB	HKPolyU-DB	0.58
SDUMLA-HMT-DB	HKPolyU-DB	1.80

**Table 7:** Comparisons of EERs of the proposed method and other domain adaptation methods in case of training with SDUMLA-HMT-DB and testing with HKPolyU-DB (unit: %).

Method	EER
No domain adaptation	1.80
StarGAN-v2 [38]	1.34
ComboGAN [39]	2.77
CycleGAN (proposed method)	0.85

**Table 8:** Comparisons of EER by the state-of-the-art methods and the proposed method in case of training with SDUMLA-HMT-DB and testing with HKPolyU-DB (unit: %).

Method	EER
Huang et al. [40]	9.46
Miura et al. [41]	6.49
Liu et al. [42]	5.01
Gupta et al. [43]	4.47
Miura et al. [44]	4.45
Dong et al. [45]	3.53
Liu et al. [46]	1.47
Xi et al. [47]	1.44
Joseph et al. [48]	1.27
Proposed method	0.85

**Table 9:** Comparisons of EER with same domain and cross-domain environment without our domain adaptation method (unit: %).

Training of Finger-Vein Recognition Model	Testing of Finger-Vein Recognition Model	EER
SDUMLA-HMT-DB	SDUMLA-HMT-DB	2.17
HKPolyU-DB	SDUMLA-HMT-DB	4.42

**Table 10:** Comparisons of EERs of the proposed method and other domain adaptation methods in case of training with HKPolyU-DB and testing with SDUMLA-HMT-DB (unit: %).

Method	EER
No domain adaptation	4.42
StarGAN-v2 [38]	4.43
ComboGAN [39]	8.96
CycleGAN (proposed method)	3.40

**Table 11:** Comparisons of EER of the state-of-the-art methods and proposed method in case of training with HKPolyU-DB and testing with SDUMLA-HMT-DB (unit: %).

Method	EER
Jalilian et al. [18]	3.57
Pham et al. [49]	8.09
Miura et. al. [44]	5.46
Miura et al. [41]	4.54
Yang et al. [50]	3.96
CycleGAN (proposed method)	3.40

**Table 12:** Comparisons of the previous and proposed methods for hand-based biometrics.

Categories	Considering the Cross-Domain Problem	Method	Modality	Advantage	Disadvantage
Non-training-based	No	Wide line detector and pattern normalization [40]	Finger-vein	Simple and computationally efficient than training-based method	Performance is not good compared to training-based method
		Maximum curvature points [41]			
		Minutiae matching [42]			
		Multi-scale matched filter [43]			
		Repeated line tracking [44]			
		Personalized best patches map [45]			
		Superpixel-based [46]			
		Discriminative binary codes [47]			
		Fuzzy rule-based [48]			
		Local binary pattern [49]			
		Tri-branch vein structure [50]			
	Yes	Dimension reduction and orientation coding algorithm [7]	Palmprint		
		SIFT [8]	Dorsal hand-vein		
		Improved SIFT [9]			
		BGP and Gabor-HoG [10]	Fingerprint		
		Least square-based domain transformation function [11]			
Training-based	No	VGG-16 and CNN [20]		Preprocessing is not required	No consideration about the heterogeneous data problem
		Patch-based MobileNet [21]			
		CGAN [19]			Does not show good performance in cross-sensor environments
		FCN [18]	Finger-vein	Using compact information on recognition stage increases generality	Unreliable label data were used
	Yes	FLDA [12]	Face and fingerprint	Simple method for domain adaptation	Needs multiple modality data from same people
		Universal material translator wrapper [13]	Fingerprint	Uses a simple style transfer network	Generated images cannot deal with level 3 features
		DeepDomainPore network [14]		Can exploit level 3 features using low-resolution input	Long preprocessing time and ground truth required for source data
		PalmGAN [15]	Palmprint	Automatically generates label data for target domain	- Long preprocessing time and ground truth required for source data Segmentation method is unstable
		Auto-encoder [16]		Automatically generates label data for target domain Simple method for domain matching with good matching performance	
		DeepScatNet and RDF [17]	Finger-selfie		
		CycleGAN-based (Proposed method)	Finger-vein	High performance for domain adaptation Does not need ground truth for source data	Intensive training for CycleGAN is necessary

## Data Availability

Trained models with algorithm can be available upon reasonable request according to the instructions in [25].
